# The Anti-Aging Mechanism of Metformin: From Molecular Insights to Clinical Applications

**DOI:** 10.3390/molecules30040816

**Published:** 2025-02-10

**Authors:** Ting Zhang, Lijun Zhou, Meagan J. Makarczyk, Peng Feng, Jianying Zhang

**Affiliations:** 1 Department of Orthopaedic Surgery, University of Pittsburgh, Pittsburgh, PA 15213, USA; 2 School of Public Health, Xinjiang Medical University, Urumqi 830011, China; 3 Department of Bioengineering, University of Pittsburgh, Pittsburgh, PA 15213, USA; 4 School of Medicine, China Academy of Chinese Medical Sciences, Beijing 100700, China

**Keywords:** metformin, anti-aging, mitochondrial function, inflammation, clinical trials, epigenetic regulation, nutrient sensing, autophagy

## Abstract

Aging represents a complex biological phenomenon marked by the progressive deterioration of physiological functions over time, reduced resilience, and increased vulnerability to age-related diseases, ultimately culminating in mortality. Recent research has uncovered diverse molecular mechanisms through which metformin extends its benefits beyond glycemic control, presenting it as a promising intervention against aging. This review delves into the anti-aging properties of metformin, highlighting its role in mitochondrial energy modulation, activation of the AMPK-mTOR signaling pathway, stimulation of autophagy, and mitigation of inflammation linked to cellular aging. Furthermore, we discuss its influence on epigenetic modifications that underpin genomic stability and cellular homeostasis. Metformin’s potential in addressing age-associated disorders including metabolic, cardiovascular, and neurodegenerative diseases is also explored. The Targeting Aging with Metformin (TAME) trial aims to provide key evidence on its efficacy in delaying aging in humans. Despite these promising insights, significant challenges persist in gaining a more comprehensive understanding into its underlying mechanisms, determining optimal dosing strategies, and evaluating long-term safety in non-diabetic populations. Addressing these challenges is crucial to fully realizing metformin’s potential as an anti-aging therapeutic.

## 1. Introduction

**Figure 1:**
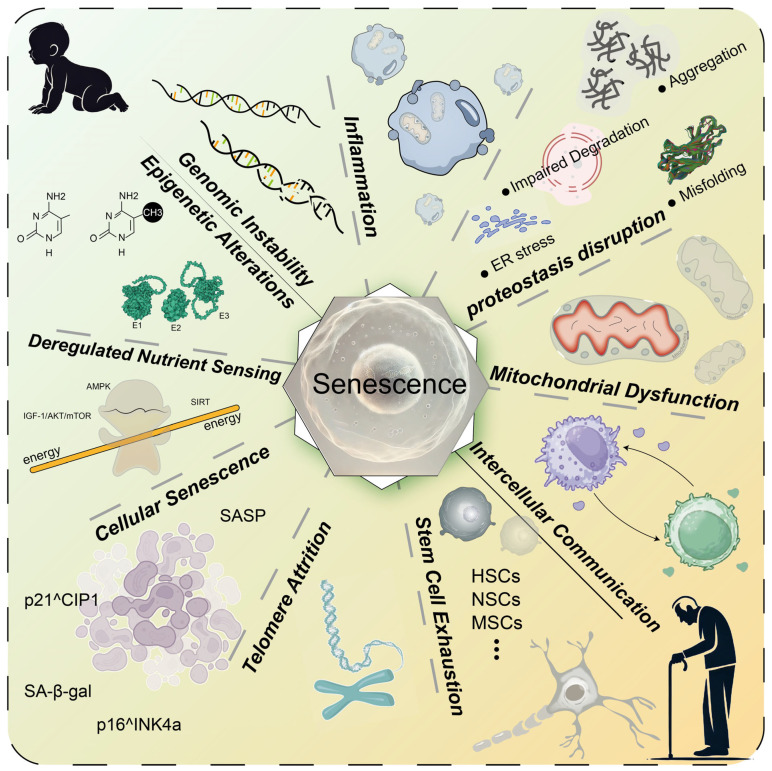
Key mechanisms of aging. Aging involves various molecular and cellular mechanisms, including genomic instability, inflammation, proteostasis disruption, mitochondrial dysfunction, altered intercellular communication, stem cell exhaustion, telomere attrition, cellular senescence, deregulated nutrient sensing, and epigenetic alterations. These interconnected mechanisms collectively drive the aging process and contribute to the increased risk of age-related diseases. HSCs: hematopoietic stem cells; NSCs: neural stem cells; MSCs: mesenchymal stem cells; ER: endoplasmic reticulum; E1: E1 ubiquitin-activating enzyme; E2: E2 ubiquitin-conjugating enzyme; E3: E3 ubiquitin ligase.

Aging is a universal natural phenomenon, and anti-aging therapies have consistently been a focal point of research throughout history. From a healthcare perspective, aging can increase the prevalence of diseases such as cardiovascular disease, neurodegenerative diseases, and metabolic disorders [1]. This complex process encompasses interconnected mechanisms, including genomic instability, telomere depletion, epigenetic changes, proteostasis disruption, mitochondrial dysfunction, nutrient-sensing impairments, cellular senescence, and persistent inflammation (Figure 1) [2,3]. These pathways collectively drive the decline in resilience and the onset of age-related diseases (ARDs).

Metformin, a popular drug used to treat type 2 diabetes mellitus (T2DM) [4], has demonstrated significant potential beyond glycemic control [5]. Emerging evidence suggests that metformin can modulate key aging-related processes, including energy regulation, inflammation, and autophagy, thereby delaying aging and mitigating ARDs [6,7,8]. Studies using diverse cell and animal models have supported its anti-aging effects [9,10]. Given its potential to extend lifespan and improve health outcomes, metformin is the focus of the first major clinical trial for anti-aging interventions—the Targeting Aging with Metformin (TAME) trial initiative [11]. The trial represents a critical step toward recognizing aging as a therapeutic target and exploring metformin as a foundational intervention for extending healthspan.

This review examines metformin’s role in elucidating molecular mechanisms of aging and fostering clinical advancements in anti-aging medicine. It emphasizes the pressing need for novel interventions that promote healthy aging while addressing challenges such as dosing, safety, and efficacy in diverse populations.

## 2. Molecular Mechanism of Metformin on Anti-Aging Effects

Metformin has garnered significant attention for its ability to target various signs of aging, including mitochondrial dysfunction, dysregulated nutrient sensing, inflammation, and epigenetic alteration. This section delves into the molecular underpinnings of metformin’s anti-aging actions and its potential applications in mitigating age-related diseases.

### 2.1. Mitochondrial Function and Energy Metabolism Regulation

Mitochondria are critical for energy generation and cellular balance [12]. Mitochondrial DNA (mtDNA) damage is closely linked to aging and cellular function and viability. Such damage leads to reduced energy generation, increased cellular stress, and the activation of apoptosis, ultimately accelerating the aging process and affecting cell longevity [13]. These mitochondrial changes are key drivers of inflammation, oxidative stress, and functional decline during aging [12]. Metformin acts on mitochondria by partially suppressing complex I of the electron transport chain, resulting in an increased AMP/ATP ratio. This triggers the activation of AMP-activated protein kinase (AMPK), which restores energy balance and promotes mitochondrial health [14].

A study utilized a 1-Methyl-4-phenyl-1,2,3,6-tetrahydropyridine (MPTP)-induced mouse model of Parkinson’s disease and an H_2_O_2_-induced astrocyte senescence model, with metformin intervention. Results demonstrated that metformin ameliorates mitochondrial function and delays astrocyte senescence via the Mfn2-cGAS signaling pathway, thereby mitigating neurodegenerative pathology [15]. AMPK modulates the methylation status of histone H3K79, thereby influencing NAD-dependent protein deacetylase sirtuin-3 (SIRT3) transcriptional regulation. As a mitochondrial deacetylase, SIRT3 plays a crucial role in regulating mitochondrial function and biogenesis [16]. Therefore, AMPK promotes mitochondrial biogenesis and delays cellular senescence by increasing SIRT3 expression through H3K79 methylation, ultimately mitigating age-related vascular dysfunction [16].

The effects of metformin on thymic degeneration were also investigated by establishing a mouse model of thymic atrophy. Histological analysis, immunofluorescence staining, mitochondrial function assays, and molecular biological techniques were employed to assess thymic cell proliferation, apoptosis, and energy metabolism. The results demonstrated that metformin ameliorates thymic degeneration by enhancing mitochondrial function, suppressing oxidative stress, and reducing thymic cell apoptosis. These findings suggest the potential application of metformin in anti-aging interventions through mitochondrial function modulation [17].

A recent study established an aged mouse skin wound model and applied metformin-engineered extracellular vesicles (Met-EVs) for therapeutic intervention. Metabolomic analysis, histological evaluation, and functional assays were performed to assess the effects of Met-EV on wound healing. The results demonstrated that Met-EVs facilitate the repair of aged wounds by modulating energy metabolism [18].

Aging is a modifiable phenomenon regulated by an array of signaling pathways, one of which involves reactive oxygen species (ROS)-mediated oxidative stress [19]. ROS, which are generated by diverse intracellular compartments, are primarily produced through the mitochondrial electron transport chain (ETC) [20]. When present in excessive amounts, these radicals induce oxidative alterations that compromise essential macromolecules—such as lipids, DNA, mitochondrial DNA, and proteins—thereby accelerating cellular deterioration [21]. Notably, the antidiabetic agent, metformin, curbs mitochondrial ROS generation by selectively inhibiting reverse electron flow through complex I, highlighting its potential role in mitigating oxidation-driven aging [22]. By improving mitochondrial efficiency, metformin reduces ROS production and enhances cellular resilience against oxidative stress, a hallmark of aging [23].

### 2.2. Nutrient Sensing Pathways

Nutrient sensing is a central mechanism that coordinates nutrient uptake and signaling, thereby modulating numerous biochemical and metabolic pathways critical for organismal homeostasis [21]. Key contributors to this process include the insulin/insulin-like growth factor (IGF)-signaling (IIS) and mechanistic target of rapamycin (mTOR) pathways [24], each capable of detecting variations in nutrient availability or growth factors [25]. The IIS pathway governs energy and protein metabolism, as well as the proliferation and differentiation of insulin/IGF-1-responsive cells. Reduced IIS activity is linked to the prolongation of lifespan, whereas its overactivation hastens aging [26]. As an antidiabetic agent, metformin can effectively lower insulin and IGF-1 levels while improving insulin sensitivity, thereby influencing this pathway [26]. Meanwhile, the mTOR pathway plays a vital role in regulating nutrient signaling and cell growth, in part through IGF-1 mechanisms, and has also been implicated in accelerated aging [27]. Evidence indicates that inhibiting either the IIS or mTOR pathway extends both median and maximal lifespans in model organisms such as Drosophila melanogaster [28] and mice [29].

Moreover, growing data suggest that metformin engages multiple nutrient-sensing cascades associated with aging, producing effects reminiscent of calorie restriction (CR), including delayed aging, enhanced longevity, and reduced incidence of age-related diseases [30]. Hence, these nutrient-sensing regulators collectively orchestrate fundamental cellular processes shaping lifespan and healthspan, underscoring metformin’s therapeutic promise in this context [31]. Although it is well documented that metformin affects nutrient sensing pathways, recent findings suggest that its impact is more likely to involve phosphorylation-dependent modifications rather than changes in protein abundance [32]. Further investigations are needed to clarify how these phosphorylation events influence cellular processes and contribute to metformin’s anti-aging effects.

### 2.3. AMPK-mTOR Pathway and Autophagy Activation

AMPK and the mechanistic target of rapamycin complex 1 (mTORC1) are pivotal players in maintaining cellular energy balance by detecting cellular ATP and nutrient levels, including glucose and amino acids [33]. These pathways act as key regulators of autophagy, a critical process that facilitates cellular maintenance by removing damaged materials and supplying energy sources and building blocks. The AMPK–mTOR axis collaborates with autophagy to meticulously adjust metabolic processes, ensuring proper cellular function under stressful conditions [34]. By activating AMPK, metformin inhibits the mTOR pathway, a key modulator of cellular anabolic progression, proliferation, autophagy, and aging [35].

Yu et al. used human foreskin fibroblasts and Kunming mice to investigate metformin’s protective effects against ultraviolet A (UVA)-induced skin aging. They found that metformin treatment reduced UVA-induced cell viability loss, skin aging markers, and activation of the PI3K/AKT/mTOR signaling pathway. Additionally, metformin alleviated mitochondrial oxidative stress and decreased mitophagy. In vivo, metformin administration significantly improved skin roughness, epidermal thinning, and collagen degradation caused by UVA exposure. These findings suggest that metformin exerts anti-photoaging effects by inhibiting mitophagy and the PI3K/AKT/mTOR pathway [36].

Metformin’s inhibition of mTOR is achieved through AMPK activation and the IIS pathway, which also leads to the activation of Unc-51-like autophagy-activating kinase 1 (ULK1) via dephosphorylation [37]. ULK1 is indispensable for instigating and coordinating early phagophore formation, orchestrating macrophage, and generating autophagosomes required for effective autophagy [38]. Through mTOR inhibition, AMPK prevents the suppression of ULK1, thereby maintaining autophagic flux and cellular homeostasis [39]. Autophagy defects and disruptions in the AMPK-mTOR-ULK1 signaling cascade impair autophagy, contributing to cellular degeneration, accelerated apoptosis, and tissue aging [40]. For instance, impairments in this pathway have been implicated in age-related deterioration of the hearing cortex, emphasizing the critical role of AMPK-mTOR signaling in maintaining cellular integrity and delaying aging [41]. In senescent cells, mTOR activity is elevated and plays a crucial role in maintaining the senescent state, including the senescence-associated secretory phenotype (SASP) [42]. Interestingly, autophagy is also upregulated in these cells, providing amino acids that activate mTORC1, which in turn supports SASP synthesis. This indicates a complex, context-dependent relationship where autophagy can both suppress and activate mTOR, contributing to the maintenance of cellular senescence [42].

### 2.4. Inflammation and Cellular Senescence Inhibition

A hallmark of aging is pervasive chronic inflammation. The secretory phenotype of senescent cells promotes the production of an ensemble of bioactive factors including cytokines, growth factors, chemokines, and matrix-degrading enzymes [43]. SASP exacerbates inflammation, alters tissue microenvironments, and accelerates the progression of aging-related dysfunctions. Concomitantly, persistent inflammation expedites immune cell senescence, diminishing immunological capabilities and thwarting the clearance of senescent cells and inflammatory mediators, thereby perpetuating the cycle of inflammation and aging. Consequently, inflammation is increasingly recognized as an intrinsic driver of aging, suggesting that its abatement may present an effective anti-aging intervention [12,44,45]. In parallel, immune cells—central to the regulation of cellular senescence—have garnered considerable research interest [46,47], particularly as they undergo significant functional impairments with advancing age, compromising their capacity to eliminate senescent cells and control chronic inflammation. Hence, modulating immune function emerges as a promising strategy to delay aging.

As mentioned previously, metformin exerts its anti-inflammatory effects through multiple pathways, particularly via the regulation of AMPK/Nuclear Factor-κB (NF-κB), AMPK/mTOR, and ROS. The anti-inflammatory mechanisms of metformin make it a strong candidate drug for treating inflammatory diseases and delaying inflammation-related aging processes [48]. Further assessing the relationship between AMPK signaling and metformin, Yang et al. examined the impact of metformin on thyroid-associated ophthalmopathy (TAO) by utilizing orbital fibroblasts obtained from TAO patients and healthy controls. They found that metformin activates the AMPK signaling pathway and inhibits mTOR activity, thereby reducing the expression of inflammatory and fibrotic markers, suggesting that AMPK may be a potential therapeutic target for TAO [49].

Nikolajczyk et al. examined the effects of metformin on mitochondrial function, autophagy, and aging-related inflammation. Using cellular and murine models treated with varying doses of metformin, the study evaluated mitochondrial function by assessing membrane potential, oxidative phosphorylation efficiency, and ATP production. Autophagy-related protein expression was analyzed via fluorescence microscopy and Western blot, while inflammatory cytokine levels were quantified using enzyme-linked immunosorbent assays (ELISAs) and quantitative real-time polymerase chain reaction (qPCR). The results demonstrated that metformin enhanced autophagy, promoting the clearance of damaged mitochondria and preserving mitochondrial integrity. It increased mitochondrial membrane potential, oxidative phosphorylation efficiency, and ATP production, indicating improved mitochondrial function. By restoring mitochondrial health, metformin reduced age-associated inflammatory cytokine levels and mitigated chronic inflammation [50]. These findings collectively highlight metformin’s potential to extend healthspan in animal models [50]. Additionally, in adipose tissue, metformin suppresses inflammation and prevents adipocyte aging by modulating cell cycle pathways, even under obese conditions [51]. Similarly, in human periodontal ligament cells, metformin-induced autophagy inhibits oxidative stress and cellular aging [52]. Furthermore, metformin prevents endothelial cell senescence by upregulating Sirtuin 1 (SIRT1) activity, which enhances vascular integrity [53]. It also mitigates macrophage senescence and SASP by suppressing NLR Family CARD Domain Containing 4 (NLRC4) phosphorylation, which is a driver of cellular senescence [54].

### 2.5. Epigenetic Regulation

Epigenetic factors orchestrate the complex regulatory network underlying inflammatory disorders by shaping gene expression patterns without altering the DNA sequence. These modifications influence fundamental cellular activities, including senescence, thus playing a pivotal role in the link between aging and chronic inflammation [55]. Indeed, epigenetic reprogramming and cellular senescence represent two key hallmarks of physiological aging [56], offering potential therapeutic targets to mitigate age-associated diseases, preserve functional capacity, and optimize healthspan. Emerging evidence suggests that metformin exerts anti-aging effects by influencing both transcriptional and post-transcriptional activities through various epigenetic mechanisms [57].

One key target of metformin is ten-eleven translocation 2 (TET2), which is associated with DNA demethylation. Metformin has been shown to enhance TET2 stability, preventing aberrant DNA methylation patterns that can lead to genomic instability in aging cells. This stabilization supports genomic integrity and reduces the risk of age-associated diseases [21]. In addition, metformin influences histone modifications through its effects on histone deacetylases (HDACs). HDAC dysregulation is associated with conditions like sarcopenia and neurodegenerative disorders. Metformin’s ability to modulate HDAC activity suggests its role in restoring epigenetic balance in aging cells [58]. Wang et al. collected peripheral blood samples from 32 diabetic patients and assessed epigenetic age acceleration using the Hannum, Horvath, and DNAm PhenoAge epigenetic clocks. The results showed that patients taking metformin experienced significantly slower epigenetic age acceleration according to the Horvath and Hannum clocks, suggesting that metformin may have a role in slowing epigenetic aging [59].

Jeon et al. explored the protective role of metformin on mesenchymal stromal cells (MSCs) within a chronic kidney disease (CKD) model. The study revealed that metformin reduces DNA damage and suppresses aging markers by activating the AMPK pathway and decreasing oxidative stress. Furthermore, metformin pretreatment significantly enhanced the regenerative capacity and survival rate of MSCs in the CKD model. These findings suggest that metformin exerts its effects by inhibiting aging and preserving mitochondrial function [60]. Beyond DNA and histone methylation, metformin upregulates the expression of DICER1, an enzyme critical for microRNA (miRNA) processing and downstream miRNAs involved in regulating cellular senescence and aging phenotypes [61]. Clinical studies have revealed that metformin modulates aging-related pathways within skeletal muscle and subcutaneous fat tissue, particularly through the regulation of miRNA-29b expression in older individuals [62]. These miRNAs influence both metabolic and non-metabolic aging processes, highlighting metformin’s extensive role in epigenetic regulation.

## 3. Systemic Anti-Aging Effects of Metformin

**Figure 2:**
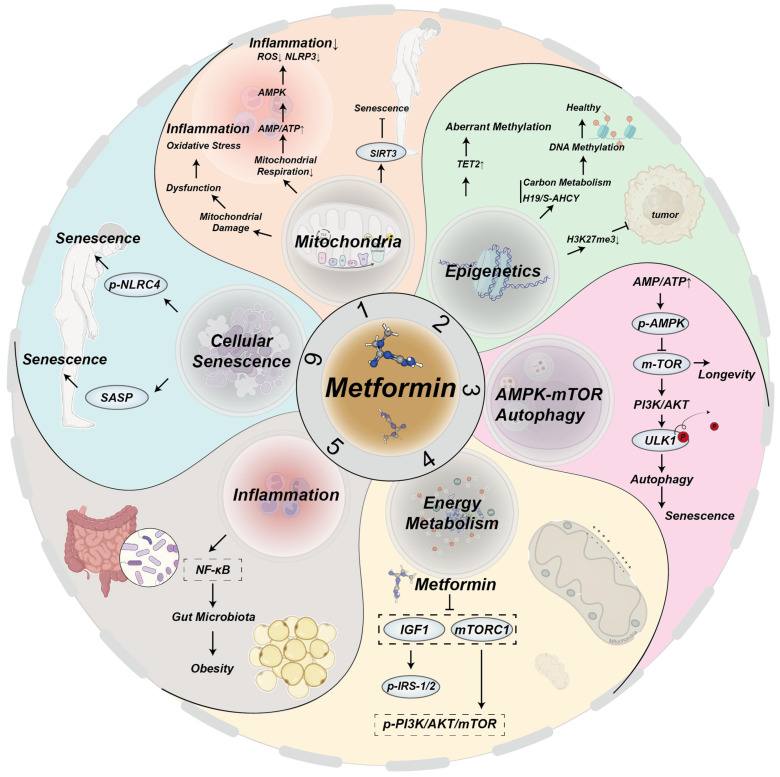
Mechanisms by which metformin targets aging and age-related diseases.

Metformin has demonstrated its effectiveness not only in the treatment of diabetes but in many aging-related diseases, including metabolic health and maintenance of glucolipid homeostasis, cardiovascular disease, neurodegenerative diseases, and oncology. The mechanisms by which metformin combats aging and age-related diseases are illustrated in Figure 2.

Metformin mitigates aging and age-related diseases by targeting hallmarks of aging through multiple signaling pathways:

(1) Mitochondrial damage leads to mitochondrial dysfunction, oxidative stress, and inflammation. A decrease in mitochondrial respiration increases the ratio of AMP/ATP, activating AMPK, which in turn blocks mitochondrial ROS production and reduces NOD-like receptor family pyrin domain containing 3 (NLRP3) and inflammation [63,64,65]. Metformin upregulates SIRT3, delaying aging [16];
(2) Metformin enhances TET2 stability, preventing aberrant methylation that causes genomic instability in the elderly [21]. Through mitochondrial one-carbon metabolism and the H19/S–adenosylhomocysteine hydrolase axis, metformin promotes global DNA methylation and regulates epigenetic gene expression to support health [66]. Additionally, metformin reduces H3K27me3 and inhibits ovarian cancer [67];
(3) Metformin senses intracellular ATP/AMP ratios, activating the AMPK and inhibiting the mTOR pathways to delay aging [35]. It induces mTOR inhibition and PI3K/AKT activation, resulting in ULK1 dephosphorylation, autophagy induction, and aging mitigation [37];
(4) Metformin influences energy metabolism and aging primarily through IGF-, mTORC1-, AMPK-, and SIRT1-mediated signaling pathways [5]. It decreases IGF-1 levels, thereby inhibiting phosphorylation of IRS-1/2 and the PI3K/AKT/mTOR signaling pathways [68]. Additionally, metformin upregulates IRS/PI3K/AKT signaling, alleviating aging [69];
(5) Metformin inhibits NF-κB signaling induced by pro-inflammatory factors [70];
(6) Senescent cells produce SASPs factors, which contribute to the aging process [12]. Metformin downregulates NLRC4 phosphorylation, inhibiting macrophage senescence.

### 3.1. Metabolic Health and Maintenance of Glycolipid Homeostasis

The global prevalence of diabetes has risen significantly with aging, and people with diabetes are at higher risk for age-related complications, including cognitive disability, Alzheimer’s disorder, cardiovascular disorders, and vision loss [12]. This association underscores diabetes as a potential accelerant of aging-related processes [12]. Metformin, a widely used antidiabetic medication, enhances metabolic health by decreasing plasma glucose levels, reducing gut intake of glucose, inhibiting hepatic glucose production, and increasing tissue sensitivity to insulin [4]. Obesity is a complex chronic condition often co-occurring with metabolic disorders such as T2DM, fatty liver disorder, and cardiovascular conditions. The comparison of established aging metrics with common obesity risk factors revealed that the numerous hallmarks that drive aging pathologies also characterize the disease processes of obesity [71]. Ample evidence indicates that obesity may accelerate the aging process [72].

Beyond glycemic control, metformin has shown efficacy in addressing obesity and its associated metabolic dysfunctions. A meta-analysis explored the effects of metformin on obese patients, selecting randomized controlled trials (RCTs) that compared metformin with placebo or other interventions in obese participants. The results indicated that metformin showed measurable benefits in weight loss and metabolic improvement in obese individuals, even among those without a diagnosis of type 2 diabetes.

Additionally, the study found that metformin effectively improves common metabolic disorders associated with obesity by enhancing insulin sensitivity reducing the risk of progressing to type 2 diabetes [73].

Metformin has the potential to treat obesity by targeting brown adipose tissue (BAT) to regulate energy metabolism. By activating the AMPK pathway and boosting heat generation, metformin enhances BAT functionality, facilitating energy expenditure and fat oxidation [74]. These findings indicate that metformin could be a promising approach for weight management and improving metabolic health [74]. Aging and obesity are strongly linked to the reduced regenerative potential of stem cells derived from adipose tissue. Metformin was indicated to improve human adipose stem cell functionality by inhibiting mTOR and extracellular signal-regulated kinase (ERK) signaling pathways, which promote mechanisms that are implicated in stem cell senescence and dysfunction [75]. Additionally, metformin’s ability to target stress-induced adipose stromal cell senescence may ameliorate age-related adipose tissue dysfunction, further supporting its role in combating the metabolic consequences of aging [76].

### 3.2. Cardiovascular System Protection

Age-related cardiovascular diseases encompass various conditions, including coronary heart disease and heart failure. Age-related vascular dysfunction and oxidative stress contribute significantly to the progression of cardiovascular diseases. Growing evidence suggests that metformin provides robust cardioprotective effects, positioning it as a potential therapeutic for preventing and managing age-associated cardiovascular diseases [77,78]. Findings from multiple clinical trials, such as the United Kingdom Prospective Diabetes Study (UKPDS), have demonstrated metformin’s ability to lower cardiovascular risk for individuals with T2DM. In newly diagnosed overweight or obese T2DM patients, early intensive metformin therapy reduced all-cause mortality by 20% and the risk of myocardial infarction by 31% compared to conventional treatment. Notably, these cardioprotective benefits persisted over time, with the legacy effects extending throughout the patients’ lifetimes [79]. Metformin’s cardiovascular benefits extend beyond glycemic control. By decreasing myocardial oxygen consumption, metformin alleviates cardiac workload and improves myocardial efficiency [80]. Additionally, metformin has been shown to significantly reduce the left ventricular mass index (LVMI) and in situ systolic blood pressure in patients suffering from coronary artery disease. These effects are complemented by its ability to attenuate oxidative stress, a critical factor in cardiovascular disease etiopathogenesis [81].

### 3.3. Nervous System and Cognitive Function

Aging has a significant impact on the progression of neurodegenerative conditions. Study finds that metformin may offer neuroprotective effects, particularly in mitigating the progression of Alzheimer’s disease (AD) and improving cognitive function [82]. From a neurological perspective, its mechanisms of action span across multiple pathways, including the modulation of glucose metabolism, reduction in neuroinflammation, and enhancement of cholinergic activity, which is critical for maintaining cognitive performance. In models of scopolamine-induced memory impairment in aging animals, metformin has been shown to improve cholinergic function [83]. A large meta-analysis has further proved that T2DM participants using metformin were at a decreased risk of suffering from AD than participants not using metformin [84]. Similarly, prospective research supports the view that metformin decelerates cognitive loss among elderly diabetic individuals, highlighting its potential for preserving brain function [85]. Despite these promising findings, the effectiveness of metformin in treating AD remains a subject of debate. In younger mice, long-term metformin treatment (1–2 years) improved attention, self-control, and cognitive flexibility. However, in older mice, prolonged metformin treatment impaired memory retention and learning, particularly in models already exhibiting AD pathology [86]. These results indicated that the influence of metformin for cognitive function likely differed according to the age and baseline neurodegenerative state of the individual [86]. Clinical evidence from human studies aligns with these findings. In a cohort study that compared the risk of dementia in two groups of elderly T2DM participants, it was found that metformin lowered the risk of dementia by 66% compared to the untreated group. Additionally, they discovered that higher doses of daily and accumulated metformin treatment were related to a lower incidence rate of dementia. Results from this study led to the conclusion that there is an important link between the dosage of metformin used and the individual’s condition [87].

### 3.4. Tumorigenesis and Immune Regulation

Despite its well-established treatment for T2DM, metformin has also been used for its anti-cancer effect and ability to modulate the immune microenvironment. Epidemiological studies have consistently reported its potential to decrease the risk of cancer growth and progression [88,89,90,91]. At the molecular level, metformin exerts its anti-carcinogenic effects by regulating key pathways, including NF-κB activation and epithelial–mesenchymal transition (EMT). For example, metformin has been shown to inhibit the carcinogenic potential of 50 Hz magnetic fields in fibroblasts of aged mice by suppressing NF-κB activity and reducing EMT, thereby mitigating tumorigenesis [92].

In preclinical and clinical settings, metformin continues to show promise. For instance, in studies using normal and T-cell-deficient SCID mice, metformin reduced the growth of various solid tumors by increasing the infiltration of CD8^+^ tumor-infiltrating lymphocytes (TILs). These TILs were associated with the generation of pro-inflammatory cytokines, such as interleukin-2 (IL-2), tumor necrosis factor-α (TNF-α), and interferon-gamma (IFN-γ), suggesting metformin’s effect for activating anti-tumor immune responses [93].

In aging populations, metformin has also shown promise in addressing ovarian fibrosis, which can increase the incidence of ovarian cancer in postmenopausal women [94]. An in vivo study using elderly C57/lcrfa mice demonstrated that metformin treatment reduced CD8^+^ T cell infiltration, the CD206^+^:CD68^+^ cell ratio, and markers of pro-inflammatory and pro-tumorigenic immune activity. These findings suggest metformin’s potential to ameliorate age-related fibrosis and reduce cancer risk [94].

Clinical evidence further supports metformin’s role in modulating the tumor immunological microenvironment. In human esophageal squamous cell carcinoma, metformin enhanced the levels of cytotoxic CD8^+^ T cells while simultaneously reducing pro-tumor macrophages expressing CD163^+^ [95]. Additionally, in patients with head and neck squamous cell carcinoma, metformin increased natural killer cell infiltration and cytotoxic activity by facilitating perforin release. This effect was mediated through an AMPK-independent, mTORC1-STAT1-driven downregulation of chemokine (C-X-C motif) ligand 1, highlighting metformin’s diverse mechanisms of action in enhancing immune surveillance and cytotoxicity [96].

## 4. The Latest Progress in Preclinical Research and Clinical Trials

Numerous animal experiments and clinical tests and observational research have established the remarkable potency of metformin as a geriatric therapeutic agent. The positive effects of metformin on energy metabolism and aging are attributed to its direct targeting of these key energy sensors. Additionally, human epidemiological studies suggests that there is a link between metformin treatment and the decreased incidence of cancer [31,97,98]. This indicates that metformin effectively reduces aging-related diseases and delays aging, though its underlying mechanisms remain unresolved.

### 4.1. Anti-Aging Effects in Animal Models

To investigate effective strategies for delaying human aging, researchers have extensively utilized animal models that mimic human physiological and functional characteristics. Recent findings have validated metformin’s authentic and precise anti-aging effects in non-human primates, offering critical insights into its potential for extending human longevity [99]. Specifically, crab-eating macaques (*Macaca fascicularis*) are an ideal model for aging research as they closely resemble the genetic characteristics and physiological functions of humans. In a groundbreaking 40-month longitudinal study, middle-aged and elderly male crab-eating macaques were administered metformin and monitored using interdisciplinary techniques, including physiological assessments, medical imaging, multi-parameter blood analyses, pathological examinations across multiple tissues, and multi-dimensional omics profiling. Results indicated that long-term metformin intake significantly alleviated cortical atrophy, improved cognitive function, delayed periodontal bone loss, and decelerated aging in several organs, including the liver, heart, and lungs. Metformin also reduced biological age markers, such as the multitissue DNA methylation age, transcriptomic age, plasma protein age, and metabolite age, achieving a maximum effect equivalent to a reduction of six biological years—corresponding to approximately 18 human years. These effects were particularly pronounced in the frontal lobe and liver, highlighting metformin’s organ-specific anti-aging benefits [99].

In aged mice, metformin-mediated modulation of the gut microbiota, particularly *Akkermansia muciniphila*, was shown to regulate host inflammation-related pathways by inhibiting pro-inflammatory cytokines like IL-6. This led to improvements in cognitive function, demonstrating metformin’s potential to mitigate neuroinflammation-associated aging [100]. Similarly, studies in *Caenorhabditis elegans* revealed that metformin by itself prolonged the lifetime of wild-type (N2) and lin-35 mutant worms by modulating fertilization efficiency. Interestingly, under high-glucose conditions, lin-35 worms exhibited a significantly longer lifespan compared to N2 worms, emphasizing metformin’s interaction with metabolic pathways in lifespan regulation [24]. Beyond its systemic effects, metformin’s anti-angiogenic and neuroprotective properties have been shown to improve age-related neovascular macular degeneration in elderly models [101]. These findings further underscore metformin’s potential in addressing specific age-associated diseases while contributing to overall longevity.

### 4.2. Overview of Human Clinical Trials

The anti-aging potency of metformin in human clinical tests has garnered significant attention in recent years. Numerous small-scale studies are currently exploring its effects on aging in non-diabetic populations, marking a shift in focus from its traditional role in diabetes management to broader applications in geriatric medicine. A landmark initiative in this field is the TAME trial, authorized by the U.S. Food and Drug Administration (FDA) in 2015; this large-scale, randomized clinical trial involves participants aged 65 to 79, who are administered a standardized daily dose of metformin to evaluate its impact on lifespan and healthy life expectancy in otherwise healthy individuals. The TAME trial seeks to establish whether aging can be pharmacologically delayed, potentially redefining aging as a modifiable condition. Success in this trial would provide robust scientific evidence supporting the intervention of aging through drug therapies, while also setting a precedent for the development of new longevity-focused treatments. Moreover, the TAME trial is expected to offer valuable insights into alternative therapeutic targets and experimental frameworks, facilitating the design of future clinical studies for other potential anti-aging interventions.

Forslund et al. conducted an analysis of 784 human gut metagenomic samples to differentiate the impacts of T2DM and metformin treatment on the gut microbiota. They found that metformin treatment increases the production of health-beneficial short-chain fatty acids (SCFAs), such as butyrate and propionate [102]. A study utilizing a metabolic model of the gut microbiota in metformin-treated T2DM patients predicted and experimentally confirmed that metformin treatment increases the production of the microbial metabolite, agmatine. Agmatine was shown to regulate host lipid metabolism and potentially extend lifespan [103].

A clinical trial investigating the effects of metformin on lipid profiles in T2DM patients selected 200 participants who were treated solely with metformin (1500 mg daily). The study results showed that after 3 months of metformin treatment, fasting blood glucose levels in patients significantly decreased. Additionally, total cholesterol, triglycerides, LDL-C, and VLDL-C levels significantly decreased, while HDL-C levels significantly increased. These findings suggest that metformin not only aids in blood glucose control but also improves dyslipidemia in T2DM patients [104].

A large, long-term prospective study enrolled 5102 newly diagnosed T2DM patients, with an average follow-up of 10 years. Participants were randomly assigned to an intensive treatment group (using sulfonylureas, insulin, or metformin) or a conventional treatment group (primarily diet control). Results showed that patients receiving intensive metformin treatment had a 35% reduction in the risk of microvascular complications compared to those undergoing conventional treatment. The risk of diabetes-related death decreased by 25%, all-cause mortality was reduced by 7%, and the combined risk of fatal and nonfatal myocardial infarction decreased by 18% [105].

A systematic review of 12 randomized controlled trials (21,595 participants) and 41 observational studies (1,029,389 participants) linked metformin use to reduced cancer mortality and overall cancer incidence, yielding relative risk reductions of 35% and 31% in these two cohorts, respectively, once more underscoring metformin’s potential cancer-protective benefits [106].

Complementing these efforts, a Mendelian randomization study using data from the UK Biobank has provided genetic evidence supporting metformin’s anti-aging effects. The study revealed that metformin-induced reductions in glycosylated hemoglobin via its target gene, GPD1, are associated with a younger epigenetic age and longer leukocyte telomeres. In contrast, AMPK activation was linked solely to a younger epigenetic age. These findings suggest that metformin’s glucose-lowering properties may contribute significantly to its anti-aging effects, offering further validation of its therapeutic potential [107].

### 4.3. Challenges and Prospects in Clinical Application

In summary, metformin exhibits significant anti-aging effects in animal models, with mechanisms likely related to antioxidant gene expression networks and metabolic regulation. In the future, with further research, metformin may have a more important role in anti-aging. However, it is worth noting that despite the groundbreaking and significant nature of the TAME trial, it faces several challenges. For example, the long-term follow-up of thousands of participants represents a challenging endeavor. Additionally, the side effects and safety concerns of metformin require close attention. Finally, current research on metformin and aging is primarily based on laboratory studies and data analysis. In the future, more meaningful studies are needed to justify the anti-aging efficacy of metformin.

### 4.4. Limitations of Metformin as an Anti-Aging Therapeutic

Despite its promising anti-aging potential, metformin has notable limitations that must be considered. Common gastrointestinal side effects such as nausea and diarrhea may impact long-term medication adherence, particularly in elderly populations, and in some cases, can lead to electrolyte imbalance, including hypokalemia, hypomagnesemia, hypocalcemia, and hypophosphatemia [108,109]. Additionally, prolonged use can lead to vitamin B12 deficiency, potentially exacerbating age-related cognitive decline [110]. Metformin’s efficacy in non-diabetic individuals remains uncertain, with variations observed across different populations due to genetic and metabolic differences [111]. Furthermore, the optimal dosage and long-term safety profile for anti-aging purposes are still under investigation, with concerns about its impact on mitochondrial function and muscle health [112]. Regulatory challenges also pose hurdles, as aging is not yet classified as a treatable condition, limiting the widespread adoption of metformin for longevity interventions.

## 5. Future Directions and Outlook

The molecular mechanisms underlying metformin’s ability to delay aging and various aging-related disorders are still largely unknown. Future research is needed to better elucidate these mechanisms, which could significantly advance the development of new and effective therapeutic tactics for preventing and treating aging-related diseases with metformin.

Studies exploring the synergistic effects of metformin with other anti-aging interventions, such as caloric restriction, exercise, and combination therapies, could provide valuable insights into maximizing its therapeutic potential. Future research should first consider patients’ genetic backgrounds, drug dosages, administration methods, drug combinations, and calorie restriction to devise personalized metformin treatment strategies. Further large-scale, multicenter, double-blind, randomized, and placebo-controlled trials are essential to better elucidate metformin’s impact on aging and related diseases.

Additionally, integrating multi-omics data alongside the principles of precision and personalized medicine should be prioritized in the development of metformin-based anti-aging therapies. Attention must be given to addressing metformin’s short half-life and relatively low bioavailability to optimize its therapeutic potential, both of which are key reasons for its suboptimal efficacy. There are certain limitations in its clinical application.

Future drug delivery system designs should focus on enhancing metformin’s bioavailability, stability, and half-life while minimizing side effects to improve its clinical applicability. While metformin is already FDA-approved for oral use, its oral administration often entails adverse effects on the stomach, liver, and kidneys. Oral administration also fails to deliver effective drug concentrations to specific local tissues, such as the skin and tendons [113,114]. Our recent studies have shown that metformin-based lotion designed for topical application can directly target skin wounds to enhance wound healing and form scarless skin [114]. Our findings indicated that introducing metformin in the form of a lotion represents a novel approach that allows for the delivery of metformin to the local tissue and blood faster and more effectively than oral approaches and bypasses the digestive system. In the future, studies should focus on transdermal delivery of metformin to develop more effective strategies for metformin administration. Ultimately, advancing our understanding of metformin’s role in aging will require interdisciplinary collaboration across molecular biology, pharmacology, and clinical medicine to translate experimental findings into effective therapeutic strategies for healthy aging.

## 6. Conclusions

In conclusion, aging is an increasingly severe health issue that impacts numerous systems and organs throughout the body. Tactics for managing healthy aging and the absence of disease-related phenotypes to decrease the redundant medical burden are needed. Metformin has shown certain anti-aging effects in clinical trials, but its specific mechanisms and efficacy still necessitate additional research and validation.

## Data Availability

Not applicable.

## Abbreviations

Abbreviation	Full Form
AD	Alzheimer’s Disease
AMP	Adenosine 5‘-monophosphate
AMPK	Adenosine 5‘-monophosphate (AMP)-activated protein kinase
ARDs	Age-related diseases
ATP	Adenosine triphosphate
BAT	Brown adipose tissue
CARD	Caspase recruitment domain-containing protein
CKD	chronic kidney disease
CR	Calorie restriction
DICER1	Recombinant Dicer 1, Ribonuclease Type III
DNA	DeoxyriboNucleic Acid
ELISA	Enzyme-linked immunosorbent assay
EMT	Epithelial–mesenchymal transition
ER	Endoplasmic reticulum
ERK	Extracellular regulated protein kinases
ETC	Electron transport chain
E1	E1 ubiquitin-activating enzyme
E2	E2 ubiquitin-conjugating enzyme
E3	E3 ubiquitin ligase
FDA	Food and Drug Administration
GPD1	Glycerol-3-phosphate dehydrogenase 1 like
H3K27me3	Methylation of lysine 27 on histone H3
HDAC	Histone deacetylases
HDL-C	High-density lipoprotein cholesterol
HSCs	Hematopoietic stem cells
IFN-γ	Interferon-γ
IGF	Insulin-like growth factors
IIS	Insulin/IGF-signal
IL-2	Interleukin-2
LDL-C	Low-density lipoprotein cholesterol
LVM	Left ventricular mass
LVMI	Left ventricular mass index
Met-EV	Metformin-engineered extracellular vesicles
miRNA	MicroRNAs
miRNA-29b	MicroRNA-29b
MPTP	1-Methyl-4-phenyl-1,2,3,6-tetrahydropyridine
MSCs	Mesenchymal stem cells
mtDNA	Mitochondrial DNA
mTOR	Mammalian target of rapamycin
mTORC1	Mechanistic target of rapamycin complex 1
NF-κB	Nuclear factor kappa-B
NLR	NOD-like receptor
NLRC4	NLR Family CARD Domain Containing 4
NSCs	Neural stem cells
PI3K	Phosphatidylinositol-3-kinase
qPCR	Quantitative real-time polymerase chain reaction
RCT	Randomized controlled trial
ROS	Reactive oxygen species
SASP	Senescence-associated secretory phenotype
SCFA	Short-chain fatty acids
SIRT1	Sirtuin 1
SIRT3	Sirtuin-3
STAT1	Signal transducer and activator of transcription 1
T2DM	Diabetes mellitus type 2
TAME	Targeting Aging with Metformin
TAO	Thyroid-associated ophthalmopathy
TET2	Ten-eleven translocation 2
TILs	Tumor-infiltrating lymphocytes
TNF-α	Tumor necrosis factor-α
UKPDS	United Kingdom Prospective Diabetes Study
ULK1	UNC-51-like kinase 1
UVA	Ultraviolet radiation A
VLDL-C	Very low density lipoprotein cholesterol
